# Unpaid Informal Caregivers in South Australia: Population Characteristics, Prevalence and Age-Period-Cohort Effects 1994–2014

**DOI:** 10.1371/journal.pone.0161994

**Published:** 2016-09-20

**Authors:** Anne F. Stacey, Tiffany K. Gill, Kay Price, Rosemary Warmington, Anne W. Taylor

**Affiliations:** 1 Population Research & Outcome Studies, Discipline of Medicine, The University of Adelaide, Adelaide, South Australia, Australia; 2 School of Nursing and Midwifery, The University of South Australia, Adelaide, South Australia, Australia; 3 Carers SA, Adelaide, South Australia, Australia; Universita degli Studi di Catania, ITALY

## Abstract

**Background:**

The ongoing need for an availability of informal carers is taking on greater relevance as the global burden of disease transitions from acute fatal diseases to long term morbidity. Growing evidence suggests that extra burden on family carers may further impact on their health and ability to provide care. Important as it is to monitor the prevalence of those conditions which influence the burden of disease, it is also important to monitor the prevalence and health profiles of those who provide the informal care. The aim of this study was to demonstrate the prevalence and demographics of adult carers aged 15 and over in the state of South Australia over 20 years between 1994 and 2014.

**Methods:**

Data from nine representative, cross-sectional population surveys, conducted in South Australia, Australia were used, (total N = 26,788 and n = 1,504 carers). The adjusted prevalence estimate of carers and their demographic characteristics were determined. So as to examine whether there were any generational effects on the prevalence of carers, an Age-Period Cohort (APC) analysis was undertaken.

**Results:**

The prevalence estimates of carers increased during the two decades from 3.7% in 1994 to 6.7% by 2014. Large increases in the proportion of retired carers, those aged 70 years and over, those carers employed, and those with higher educational qualifications were observed. There were also larger proportions of respondents with a country of birth other than Australia, UK, Ireland and European counties. The APC analysis illustrated an increasing prevalence rate over each decade for carers aged 20–80 years, especially for those over the age of 60 years.

**Conclusions:**

The results illustrate changing carer characteristics and carer prevalence estimates in South Australia as new generations of carers take on the caring role. There is a need to include questions regarding informal carers within ongoing mainstream population surveys, particularly at state levels, so as to plan for their future health care and home support.

## Background

Although the concept of kinship support and filial piety has existed throughout history across most cultures, the importance of family members caring for ill or aged relatives was not adequately recognised at the level of social policy of western countries until later in the twentieth century [1–2]. Traditional expectations of family meant that the caring efforts of informal carers (unpaid caregivers) were often taken for granted [3]. Today carers are more readily recognised as a separate group in their own right. Their pivotal role in health and social support systems are acknowledged for their significant economic contribution to containing health care costs [4–5]. Defining an informal carer or family caregiver is however problematic as carers can be any age, from children to the elderly, younger than nine years old to over 90 years of age. They may care for a child or adult age person with a disability, a chronic physical or mental illness, is recovering from illness or accident or who is a frail aged person. Carers are referred to as unpaid, informal or family caregivers who provide in-home support to a family member or friend who needs assistance in their daily living activities.

Globally the ongoing need for an availability of informal carers is taking on even greater critical relevance. Firstly as life expectancy increases and the population ages; secondly as studies of global burden of disease show a transitioning from early mortality associated with acute fatal diseases to long term morbidity dominated by chronic conditions [6–7]. Not only have these phenomena resulted in higher proportions of disability and impairment across populations, but are impacting at community and individual levels [8]. For example informal family carers of all ages provide multifaceted care for children and adults with a disability, persons who are frail and aged, chronically ill partners and friends with complex and demanding physical and mental health problems [9].

Growing evidence suggests that the extra burden on carers can put them at risk of physical health and emotional stress and strain [10–12]. Therefore important as it is to track the prevalence of those conditions which influence the burden of disease, (for example cancers, cardiovascular diseases, injury as well as dementias), it is also necessary to track the prevalence, demographic profiles and health status of those who provide the informal care.

Over the past thirty years, prevalence figures of informal caregiving at the population level have emerged haphazardly across industrialised nations and more recently from developing countries [13–14]. The methodology to identify informal caregivers still remains inconsistent, with international surveys providing estimates ranging from 15% to 39% [9,15]. It is acknowledged that prevalences are affected by different definitions of informal carers thus it is difficult to compare prevalences across countries and across studies or surveys. However some larger population studies do provide an overview. For example, in 2011, 12% of the British (adult) population were identified as caregivers [16]. Canadian surveys have estimated that overall 28% of adult Canadians aged 15 or over were caregivers although rates varied considerably across the provinces [17]. In the United States (US) it was estimated that up to 25–30% of the adult population were providing care and support to family and friends, but again rates varied by state [18–19]. In both the 2009 and 2012 Australian Bureau of Statistics (ABS) Disability Ageing and Carers surveys (SDAC), approximately 12% of the Australian population was identified as providing some care while approximately a third were primary carers [20–21]. ABS limit their definition to care provided to people with disability, long-term conditions, or care for persons who are aged 60 years and over [21].

At state levels in Australia, population-based details of carer prevalence have also mostly come from data collected by the national ABS surveys. These have been conducted approximately every five to six years since 1993. In the state of South Australia (SA), the prevalence of carers has been determined through additional separate state wide Health Omnibus Surveys (HOS) which have included carer status questions in nine of the annual surveys between 1994 and 2014.

The aim of this paper is to show changes over 20 years, between 1994 and 2014, in the prevalence and demographic characteristics of adult carers aged 15 and over in the state of SA. To achieve this, the trend over the past decades was analysed. Secondly, multivariable analyses were conducted to determine the demographic characteristics of those reporting that they were carers, from three time points. Thirdly, the percentage differences for demographic and socio-economic variables were analysed across the two decades from 1994 to 2014. Lastly the age-period-cohort (APC) effects were also examined. The benefits of APC analysis allow the effects of age, period and cohort to be interpreted independently whilst taking into account a plethora of individual, societal, historical and cultural aspects [22]. Age relates to the physiological processes associated with growing older, period effects relate to particular time points with the assumption that populations are all equally affected, and cohort effects relate to experiences during particular time frames.

## Methods

The Health Omnibus Survey is a population-based cross-sectional, representative survey that has been undertaken annually or bi-annually in SA since 1990. It investigates a range of health and health service issues as requested by health related organisations and researchers in SA and beyond. The full HOS methodology has been previously described [23] but in brief, each survey is a clustered, multi-stage, systematic, self-weighting sample selected from the Adelaide metropolitan area with the remainder being drawn from those country areas with a population of 1000 or more, based on ABS Census information. Each survey is face-to-face and interviews are undertaken by trained interviewers.

### Carer questions

Informal carers are those who provide the main care in the home setting, are aged 15 years or older, and are giving ongoing personal care and assistance to dependent relatives and individuals with a chronic mental or physical illness or who are frail and aged. Providing this care is beyond that which is expected in a normal relationship [24–25]. Data pertaining to informal carer status was from selected years between 1994 to 2014. In 1994 and 1998, the carer question used was, *“Are you a carer of a dependent person*? *(A dependent person is someone who has a chronic condition that is unlikely to improve*, *for example frail aged*, *disabled etc*.*)*. In the remaining surveys (2000–2002, 2004, 2008, 2013–2014) the carer question was, *“Do you provide long-term care at home for a parent*, *partner*, *child*, *other relative or friend who has a disability*, *is frail*, *aged or who has a chronic mental or physical illness*, *where long-term care is a minimum of 6 months and may extend into years*.*”* Refer to S1 File.

### Demographic and socio-economic questions

Demographic variables included in the surveys are gender, age group, area of residence, country of birth and marital status. Socio-economic variables included educational attainment and work status. Details of the annual income for each household was obtained and the socio-economic disadvantage of neighbourhood at an environmental level (using postcode) was classified into the Socio Economic Index for Areas (SEIFA) Index of Relative Socio-Economic Disadvantage from which quintiles were determined [26]. Refer to S2 File.

### Data analysis

The survey data were weighted by age, gender and geographic locations so that the findings apply to the demographic profile of SA using either the ABS census data or the most recent estimated residential population for each year. Initially the trend in prevalence was determined using the nine years of data. Age and sex standardized prevalence estimates were also produced. For ease of interpretation data from three surveys collected ten years apart (1994, 2004 and 2014) were selected to highlight specific demographic changes. Analysis was undertaken using SPSS Statistics, Version 19 (IBM SPSS Statistics, New York, NY, USA).

For the APC analysis, the combined data from the nine years were used and an APC model was constructed using STATA Version 13 (StataCorp, College Station, TX, USA) with the ‘apcfit’ command [27]. APC analysis was chosen so as to interpret the effects of ageing, birth cohorts and time periods in relation to carer prevalence and odds ratios. In this analysis, ‘AGE’ was the self-reported age of the respondents at the time of the survey interview. ‘COHORT’ was the age subtracted from the survey year. This ranged from the oldest respondents (80 years and over) to the youngest respondents (aged 15 years). ‘PERIOD’ represents the years of data collection (1994 to 2014).

### Ethical approval

This procedure was approved by both the Research Ethics Committee of The University of Adelaide (U of A H-097-2010), and previously with South Australia Health and the South Australian Department of Health, (310/07/2012).

Participants provided informed verbal consent for each of the surveys used between 1994 and 2014 for this study. In line with other epidemiologically-based surveillance systems, verbal consent was obtained from the participant before the interview commenced. Participation in each HOS study is voluntary. An approach letter introducing HOS was sent to selected households prior to all interviews, including a brochure outlining confidentiality and privacy assurance, how the information was to be used and which organisations were involved in each survey. If the selected respondents had any queries or did not wish to participate in the survey, they were able to call a 1800 free call telephone number listed in the introductory letter.

Verbal consent was obtained by the interviewers at the time of the face-to-face interview and upon initial contact, the interviewers repeat the purpose of the survey as well as the expected length of time to complete the interview. The respondent could choose to not answer any question or section throughout the interview or could terminate participation at any time. Continued participation was taken as evidence as continued willingness to participate (implied consent) however if the participant withdrew their consent to participate at any time, the interview was terminated and no information collected.

## Results

**Fig 1:**
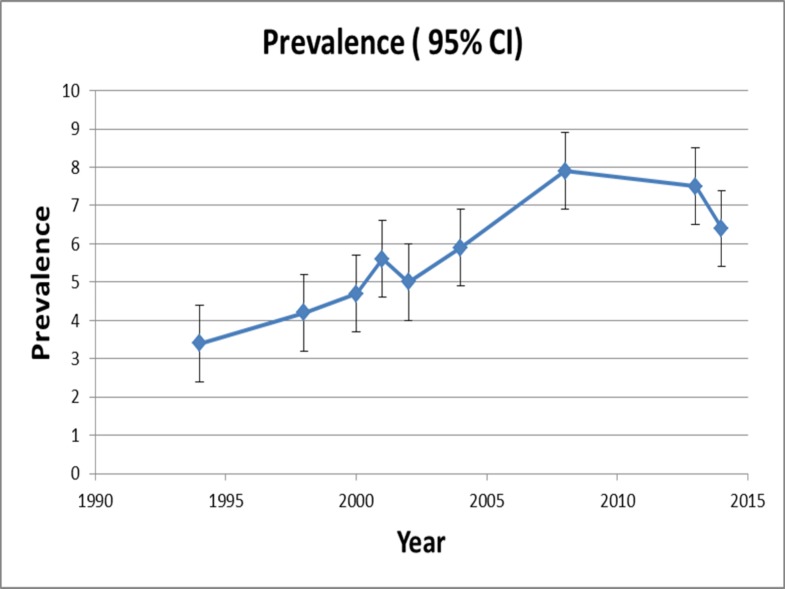
Prevalence Estimates of Adult Carers in South Australia: 1994 to 2014.

Overall, the total sample for the nine surveys was N = 26,788 (n = 1,504 were carers). The survey response rates decreased over the 20 year period from 72.4% in 1994 to 54.4% in 2014. The prevalence trend using data from nine surveys which included carer status questions is presented in Table 1 and Fig 1. In SA over the two decades from 1994 to 2014, there was almost a doubling of the prevalence of carers increasing from 3.7% 1994 to a peak of 7.9% in 2008 then declining to 6.7% by 2014.

**Table 1:** Crude & Age / Sex Standardised Prevalence Estimates of Adult Carers, South Australia 1994–2014.

Year	HOS Total N	CarersTotal n	SA Estimate Prevalence % (95% CI)	Age and Sex Standardised Prevalence Estimate % (95% CI)
**1994**	3010	104	3.4% (2.9–4.2)	3.7% (2.9–4.4)
**1998**	3010	127	4.5% (3.6–5.0)	4.6% (3.5–5.6)
**2000**	3027	141	4.7% (4.0–5.5)	4.8% (4.0–5.6)
**2001**	3037	170	5.6% (4.8–6.5)	5.9% (4.9–5.6)
**2002**	3015	154	5.0% (4.3–5.9)	5.2% (4.2–6.2)
**2004**	3015	177	5.9% (5.1–6.8)	5.9% (5.1–6.8)
**2008**	3034	239	7.9% (6.8–8.9)	7.9% (6.8–8.9)
**2013**	2908	218	7.5% (6.6–8.5)	7.5% (6.2–8.8)
**2014**	2732	174	6.4% (5.5–8.3)	6.7% (5.4–7.7)

Table 2 highlights the unadjusted and adjusted odds ratios of carers over three specific years; 1994, 2004 and 2014 by a range of demographic variables. Multivariable analysis shows that in 2014 carers were more likely to be female (OR 1.43; 95% CI 1.00–2.05, p = 0.050), be aged 50–69 (OR 1.82; 95% CI 1.11–3.01 p = 0.0.019), be born in countries other than Australia, UK or Ireland, (OR 1.82; 95% CI 1.08–3.07, p = 0.02), be never married (OR 0.47; 95% CI 0.26–0.83, p = 0.011), declare their work status as ‘home duties’ (OR 1.88; 95% CI 1.02–3.48, p = 0.043) and have annual household incomes of $20,000–40,000 (OR 2.38; 95% CI 1.29–4.02, p = 0.005).

**Table 2:** Unadjusted and adjusted odds ratios of carers over three time periods by demographic variables: Health Omnibus Survey: 1994, 2004, 2014.

		1994				2004				2014			
		unadjusted		adjusted		unadjusted		adjusted		unadjusted		adjusted	
		odds ratio	pval	odds ratio	pval	odds ratio	pval	odds ratio	pval	odds ratio	pval	odds ratio	pval
**SEX**	Male	1.00		1.00		1.00		1.00		1.00		1.00	
	Female	1.41 (0.90–2.20)	0.131	1.01 (0.56–1.81)	0.981	1.50 (1.04–2.17)	0.030	1.31 (0.87–1.98)	0.200	1.51 (1.06–2.15)	0.022	1.43 (1.00–2.05)	0.050
**AGE GROUP**	18 to 49	1.00		1.00		1.00		1.00		1.00		1.00	
	50 to 69	3.10 (1.97–4.88)	0.000	2.08 (1.22–3.54)	0.007	1.90 (1.30–2.77)	0.001	1.48 (0.95–2.29)	0.084	2.34 (1.60–3.44)	0.000	1.82 (1.11–3.01)	0.019
	70+	2.41 (1.18–4.91)	0.016	1.74 (0.69–4.35)	0.237	2.38 (1.52–3.71)	0.000	1.73 (0.93–3.20)	0.083	2.80 (1.73–4.55)	0.000	1.73 (0.79–3.77)	0.167
**AREA**	Metro. Adelaide	1.00		1.00		1.00		1.00		1.00		1.00	
	Country	1.19 (0.69–2.05)	0.521	1.07 (0.59–1.93)	0.829	1.24 (0.89–1.71)	0.197	1.13 (0.81–1.59)	0.471	1.19 (0.77–1.84)	0.427	1.14 (0.72–1.80)	0.580
**COUNTRY OF BIRTH**	Australia	1.00		1.00		1.00		1.00		1.00		1.00	
	UK/Ireland	2.26 (1.35–3.78)	0.002	1.89 (1.09–3.25)	0.022	1.30 (0.75–2.25)	0.350	1.04 (0.57–1.88)	0.901	1.66 (1.06–2.60)	0.027	1.25 (0.79–1.98)	0.343
	Other	1.09 (0.59–2.02)	0.793	0.90 (0.47–1.74)	0.750	0.73 (0.42–1.27)	0.263	0.66 (0.38–1.16)	0.149	1.54 (0.96–2.46)	0.073	1.82 (1.08–3.07)	0.026
**MARITAL STATUS #**													
	Married/defacto	1.00		1.00		1.00		1.00		1.00		1.00	
	Separated/Divorced	1.01 (0.53–1.92)	0.971	1.05 (0.53–2.05)	0.893	0.88 (0.53–1.48)	0.635	0.68 (0.39–1.19)	0.176	1.12 (0.73–1.73)	0.593	0.94 (0.56–1.58)	0.819
	Never married	0.33 (0.16–0.69)	0.003	0.62 (0.28–1.38)	0.239	0.47 (0.26–0.85)	0.012	0.59 (0.31–1.14)	0.117	0.35 (0.22–0.54)	0.000	0.47 (0.26–0.83)	0.011
**EDUCATIONAL ATTAINMENT**													
	Up to secondary	1.00		1.00		1.00		1.00		1.00		1.00	
	Trade qualifications, certificate, diploma	1.33 (0.57–3.09)	0.506	1.02 (0.61–1.69)	0.953	1.62 (0.87–3.00)	0.127	1.06 (0.71–1.60)	0.766	1.52 (0.91–2.56)	0.111	0.94 (0.67–1.32)	0.704
	Degree or higher	1.49 (0.66–3.35)	0.337	1.19 (0.48–3.00)	0.706	1.86 (1.03–3.37)	0.040	0.81 (0.43–1.53)	0.512	1.70 (1.10–2.65)	0.018	0.70 (0.42–1.17)	0.169
**WORK STATUS**													
	Employed full or part time	1.00		1.00		1.00		1.00		1.00		1.00	
	Home duties	3.37 (1.96–5.82)	0.000	2.19 (1.13–4.23)	0.020	3.32 (1.99–5.54)	0.000	2.34 (1.34–4.07)	0.003	2.80 (1.50–5.21)	0.002	1.88 (1.02–3.48)	0.043
	Retired	2.71 (1.47–4.99)	0.001	1.31 (0.58–2.96)	0.511	2.50 (1.66–3.78)	0.000	1.55 (0.92–2.61)	0.096	2.70 (1.80–4.07)	0.000	1.45 (0.84–2.50)	0.176
**HOUSEHOLD ANNUAL INCOME**													
	$40,000	1.00		1.00		1.00		1.00		1.00		1.00	
	$20-$40,000	1.72 (0.78–3.81)	0.181	1.39 (0.57–3.36)	0.465	1.82 (1.17–2.82)	0.008	1.36 (0.82–2.25)	0.234	3.44 (2.06–5.73)	0.000	2.28 (1.29–4.02)	0.005
	< $20,000	3.14 (1.59–6.17)	0.001	1.99 (0.83–4.80)	0.124	2.35 (1.59–3.46)	0.000	1.71 (0.98–2.98)	0.060	2.03 (1.13–3.64)	0.018	1.55 (0.82–2.94)	0.174
**SOCIAL DISADVANTAGE (SEIFA)**													
	Middle to highest	1.00		1.00		1.00		1.00		1.00		1.00	
	Lowest to low	1.27 (0.81–2.02)	0.299	1.06 (0.65–1.73)	0.804	1.01 (0.74–1.39)	0.928	0.88 (0.62–1.24)	0.455	1.28 (0.90–1.81)	0.161	1.20 (0.85–1.68)	0.297

# Widowed excluded

Table 3 details the percentage increase across the two decades from 1994 to 2014 by demographic characteristics. Moderate increases across the 20 years from 1994 to 2014 were seen for females (100% increase, from 4.0% (95% CI 3.0–5.4) to 8.0% (95% CI 6.6–9.5), with a 86.2% increase in males over the same time period from 2.9% (95% CI 2.1–4.0) to 5.4% (95% CI 4.0–7.4). There was a larger 120% increase for carers aged 70 years or more, from 5.0% (95% CI 3.0–8.4) to 11.0% (95% CI 8.0–14.8). It is also noted in carers aged 15–49 years, there was a 90.9% increase from 2.2% (95% CI 1.5–3.1) to 4.2% (95% CI 3.1–5.7).

**Table 3:** Prevalence of carer status by demographic variables, by three time periods: Health Omnibus Survey,- 1994, 2004, 2014.

			1994			2004			2014		% diff 1994 &2014	% diff 2004 &2014
		n	% (95% CI)	p value	n	% (95% CI)	p value	n	% (95% CI)	p value		
**Sex**	Male	43	2.9 (2.1–4.0)	0.13	70	4.8 (3.6–6.2)	0.029	73	5.4 (4.0–7.3)	0.021	86.2	12.5
	Female	61	4.0 (3.0–5.4)		107	7.0 (5.8–8.4)		110	8.0 (6.6–9.5)		100.0	14.3
**Age groups**	18 to 49	43	2.2 (1.5–3.1)	<0.001	77	4.2 (3.3–5.4)	<0.001	64	4.2 (3.1–5.7)	<0.001	90.9	0.0
	50 to 69	44	6.4 (4.8–8.5)		62	7.8 (6.1–9.8)		80	9.3 (7.3–11.8)		45.3	19.2
	70+	17	5.0 (3.0–8.4)		39	9.5 (7.0–12.9)		39	11.0 (8.0–14.8)		120.0	15.8
**Area**	Metropolitan	68	3.3 (2.6–4.1)	0.521	117	5.5 (4.6–6.6)	0.196	131	6.4 (5.2–7.9)	0.426	93.9	16.4
	Country	36	3.9 (2.4–6.1)		61	6.7 (5.3–8.6)		52	7.5 (5.3–10.6)		92.3	11.9
**Country of birth**	Australia	68	3.0 (2.3–3.9)	0.005	132	5.9 (4.9–7.1)	0.29	113	5.8 (4.6–7.3)	0.046	93.3	-1.7
	UK/Ireland	23	6.5 (4.3–9.8)		28	7.5 (4.8–11.6)		24	9.3 (6.4–13.4)		43.1	24.0
	Other	13	3.3 (1.8–5.7)		18	4.4 (2.7–7.0)		47	8.7 (5.9–12.6)		163.6	97.7
**Marital status** # Married/defacto	Married/defacto	79	4.3 (3.4–5.4)	0.003	133	7.1 (6.0–8.5)	0.003	137	8.0 (6.5–9.8)	<0.001	86.0	12.7
	Separated/Divorced	11	4.3 (2.3–7.9)		16	6.3 (4.0–9.9)		20	8.9 (6.2–12.7)		107.0	41.3
	Never married	10	1.4 (0.7–2.9)		25	3.5 (2.1–5.8)		19	3.0 (2.0–4.4)		114.3	-14.3
**Educational Attainment**	Secondary schooling	64	3.7 (2.8–4.8)	0.628	101	6.6 (5.4–8.0)	0.16	83	7.7 (6.2–9.6)	0.092	108.1	16.7
	Trade qualifications, Certificate, Diploma	33	3.3 (2.2–4.9)		61	5.8 (4.4–7.6)		68	7.0 (5.3–9.0)		112.1	20.7
	Bachelor Degree	7	2.5 (1.1–5.5)		15	3.6 (2.1–6.1)		32	4.7 (3.1–7.0)		88.0	30.6
**Work status** # Employed full or part time	Employed full or part time	33	2.1 (1.4–3.3)	<0.001	64	3.8 (2.9–5.1)	<0.001	71	4.6 (3.5–6.2)	<0.001	119.0	21.1
	Home duties	37	6.8 (4.8–9.4)		39	11.7 (8.2–16.4)		18	12.0 (6.8–20.1)		76.5	2.6
	Retired	27	5.5 (3.8–7.9)		51	9.1 (6.9–11.7)		65	11.6 (9.3–14.4)		110.9	27.5
**Household annual income** # $40,000 or more	$40,000 or more	16	1.8 (1.0–3.3)	0.004	63	4.2 (3.2–5.5)	<0.001	68	4.9 (3.5–6.8)	<0.001	172.2	16.7
	$20-$40,000	22	3.0 (1.9–4.8)		42	7.4 (5.4–10.1)		47	15.1 (11.5–19.6)		403.3	104.1
	Less than $20,000	47	5.4 (4.0–7.1)		58	9.4 (7.4–11.8)		15	9.5 (5.8–15.2)		75.9	1.1
**Index Relative Socio-disadvantage (SEIFA)**												
	Middle to highest	54	3.1 (2.3–4.1)	0.492	103	5.9 (4.8–7.1)	0.928	99	6.1 (4.9–7.5)	0.161	96.8	3.4
	Lowest low	49	3.9 (2.8–5.5)		75	5.9 (4.8–7.4)		84	7.6 (5.8–10.0)		94.9	28.8
	**Total**	**104**	**3.4 (2.8–4.3)**	** **	**177**	**5.9 (5.1–6.8)**	** **	**183**	**6.7 (5.6–8.0)**	** **	**97.1**	**13.6**

# Widowed (Marital Status: excluded

Not stated (Work Status): excluded

Not stated (Household annual income): excluded

Other socio-demographic increases over the 20 years included a 108.1% increase for carers with a secondary school level or less education, from 3.7% (95% CI 2.8–4.8) to 7.7% (CI 95% CI 6.2–9.6), however in terms of educational attainment, there was a 112.1% increase in carers with trade qualifications, certificates and diplomas, increasing from 3.3% (95% CI 2.2–4.9) to 7.0% (95% CI 5.3–9.0).

Again, large percentage increases were recorded for work status, with a 119.0% increase for employed carers (full or part time) from 2.1% (95% CI 1.4–3.3) to 4.6% (95% CI 3.5–6.2), and a 110.9% increase in those carers nominating they were retired, from 5.5% (95% CI 3.8–7.9) to 11.6% (95% CI 9.3–14.4). Annual household income of $40,000 or more showed a 172.2% increase from 1.8% (95% CI 1.0–3.3) to 4.9% (95% CI 3.5–6.8) and a 403.3% increase for annual household income of $20–40,000 was recorded, from 3.0% (95% CI 1.9–4.8) to 15.1% (95% CI 11.5–19.6).

Other demographic percentage increases included a 163.6% increase for other country of birth, increasing from 3.3% (95% CI 1.8–5.7) to 8.7% (95% CI 5.9–12.6). Carers born in Australia showed a 93.3% percentage increase from 3.0% (95% CI 2.3–3.9) to 5.8% (95% CI 4.6–7.3), however there was a smaller 43.1% increase in carers whose country of birth was UK/Ireland, from 6.5% (95% CI 4.3–9.8) to 9.3% (95% CI 6.4–13.4).

In terms of percentage differences over the most recent decade, from 2004 to 2014, highest percentage increases were seen for annual household income of $20,000–40,000 (104.1% increase) from 7.4% (95% CI 5.4–10.1) to 15.1% (95% CI 11.5–19.6). No change in the most recent decade to 2014 was found for carers in the 18–49 age group (4.2%). The only negative percentage differences occurred in the most recent decade to 2014 was for carers born in Australia (-1.7%) and carers who were never married (-14.3%).

**Fig 2:**
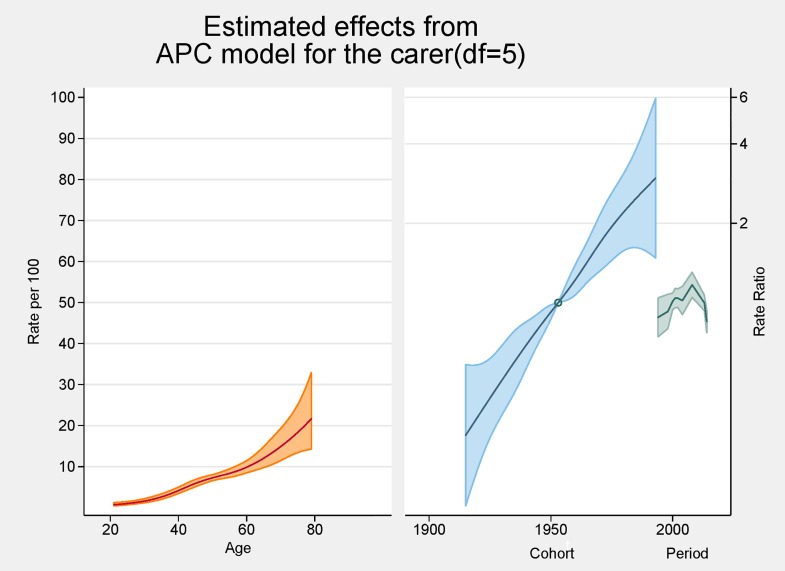
APC analysis of informal caregiving in South Australia shown as line graphs representing rates (%) and rate ratios, with 95% confidence intervals.

Fig 2 provides the results of the APC analysis. On the left axis is shown the independent effects of age (prevalence) and on the right axis the birth cohort and period effects, both using rate ratios are shown. The peak age for carers was around 80 years and showed a steady increasing rate over each decade for those aged 20–80 years. The graph indicates that the prevalence of being a carer increases, especially after the age of 60. In the cohort analysis the Baby Boomers born around 1951–2 are the reference point [= 1] and also represent the point of acceleration of risk which for the purposes of this paper, can be interpreted as each cohort’s exposure to informal caregiving. The graph also shows a higher ratio of caring in the later cohorts born mid1970s to 2000, suggesting that persons born around 1975 (Generation X) may be twice as likely to become carers, whereas persons born 1980s to 2000 (Generation Y) have a three-fold likelihood of becoming carers. The Estimated Period Effect represents the specific calendar period when the sample population were surveyed. The resulting period effects of the graph show increasing prevalence from 1994, peaking at 2008 then falling into negative effects by 2014.

## Discussion

The results indicate an inital increase in the prevalence of carers from 1994, a doubling of the proportion of carers by 2008 (3.7% to 7.9%), then a slight decline to 6.7% by 2014. Increases in proportions of carers across all ages were found, especially the 50–69 year old group and the 70 years and over age groups, a finding consistent with SA having been the state with the highest proportion of older age population in Australia [28–30]. In our surveys there was still a considerable proportion of carers whose education was secondary school or less, but there were higher prevalences of carers who had high educational attainment and tertiary training. Carers were more likely to be employed either full time or part time, but as would be expected with an ageing population, there were also more carers who had left work and retired. In terms of annual household incomes of carers which have increased over the past 20 years, much of this would be the result of inflation and many carers still remain in the low/ lowest most disadvantaged quintiles compared with the wider community.

It is well documented that women have been the traditional and dominant family caregivers in societies of all cultures [2]. Although there was a slight increase in proportions of male carers in our earlier SA survey of 2004, overall male percentage increases (86%) remained below the 100% increase of females. ABS national surveys across Australia amongst ‘all’ carers in 1998 showed there was some narrowing of the gender gap with just over a half of all carers being female. In the over 75 age group there were slightly more male primary carers. Interestingly Australian disability surveys revealed that after the age of 85, carers were frequently males caring for a disabled wife [31] The implications of this changing demographic with an increasing number of male carers in this older age group raise questions about the mix and type of services needed for the future.

There is a paucity of published literature on informal caregiving in terms of surveys applying Age-Period-Cohort methods. Earlier studies include one that explored changes in attitudes towards caring for ageing parents [32]. More general research examined trends in disability in older adult cohorts using APC [33]. Other APC studies and those specific to carers using methodology comparable to this paper have not been sourced to date, except those applied to prevalence rates of chronic disease topics such as diabetes, and obesity [34].

Our APC analysis (Fig 2) illustrates the combined impact of caregiving and ageing on several birth cohorts, which range from the older carers of pre-war generations (born early 1900s to 1945), to the Baby Boomers (born 1946–1964) and Generation X, (born 1965–1980s). The prevalence of becoming a carer showed a steady increase over each decade, especially after the age of 60. Using the APC analysis, carers’ peak age was around 80 years. Again our study indicates there has been a trend for older age carers to continue caregiving into their late 70s, 80s and beyond. Other authors have observed that the onset of caregiving peaked in late middle age and older, and that informal care could span three decades or more of adult life [35]. Of concern has been the lack of data on prevalence of those much older generations who might be providing care for a spouse, relative or an adult child with a disability. Literature is more readily available on social and health impacts on those cohorts [36–38].

Our study highlights changes in carers’ country of birth, an aspect that reflects sixty years of increased immigration to South Australia. Although our results show that there has been a 93% percent increase in carers born in Australia, by 2014 there were large percentage increases (over 163%) in carers born in countries other than Australia, UK, Ireland or Europe. This change from earlier carer profiles of the 1990s reflects the wider cultural characteristics of people (families) who have migrated to SA since the 1950s and 1960s. Australia has a rich multi-cultural heritage of people from over 200 countries. Those overseas-born persons aged 65 or over, have expanded in recent decades, doubling since 1991 [39–40]. There is potential for this cultural influence to impact on carers’ use of home based care if there are inadequate culturally appropriate respite services or facilities. Further, there could be a reluctance on the part of older persons from different cultures to accept support services creating additional difficulties for the spouse or other family carers [41].

The results of our surveys show that new generations of carers have emerged with different characteristics in areas of education and employment and this can be a challenge for many to combine with their informal caring. The higher proportion of working carers in our study, (both full time and part time), takes on greater importance because of the many Baby Boomers who are the current generation of informal carers. This transition occurred as older Pre-World War II carers, (for example, ageing parents of the early Baby Boomers), became the recipients of care over the past 20 years. This coincided with a greater emphasis on community care in Australia during the past decades, which is being further developed as new government reforms and initiatives for carers and consumers [42]. For example they focus on consumer directed care packages based on ageing in place (and in the home) and in disability ‘consumer choice and control’ through the NDIS. Both models emphasise that they are consumer directed for home based care. Current integrated support services for carers are undergoing further development.

Younger working carers, especially parent-carers, are another group that cannot be overlooked. Carers of younger children with chronic health conditions and disabilities are more likely to be subject to employment constraints. Employment for carers can be problematic and research is emerging of the negative effects on the parents who are the informal carers [43]. In general, of those carers who also work, many do manage well without adverse health changes, but Schofield concluded that ‘working carers providing high levels of care represent a vulnerable subgroup where supportive and preventive services might be focused’ [44]. In our study there was a higher proportion of working carers overall, but on a global scale, Australia has had a lower percentage of employed carers (38.4%), than other countries such as Canada, USA or the UK (55–60%) [45–48].

While the changing prevalence of carers in our study showed an increase over time, the later decline by 2014 could have been influenced by a number of factors. For example natural attrition (deaths) amongst carers from the oldest birth cohorts would have been occuring during that time. On a national scale reduced disability rates amongst both children and adults were observed which could have resulted in slightly less demand for informal carers [20]. Other factors impacting on carer numbers may be due to younger generations choosing careers over caring roles, especially with greater labour force participation of women [49]. Also it is not uncommon for people to choose to work beyond the ages of 65 and 70 years old which would lessen their availability for complex caregiving in the home. There have also been trends towards more active retirement, especially amongst baby boomers and later generations [40]. It is conjectured that some of these socio-economic factors surrounding caregiving may have been further influenced by the Global Financial Crisis (GFC) with a general reluctance to give up paid work and income during such uncertain times. Those directly affected by the GFC may have had to actively seek employment, as a priority over any caring role.

The strengths of this study are that the results provide an analysis of carer prevalence and demographics over a twenty year period. It has used population-based data using face-to-face interviews, the gold standard of surveys, and a significant number of interviews were conducted. It is therefore more generalizable to other ageing populations and includes a number of relevant demographic covariates.

Limitations of the study include definitional issues which continue to influence all caregiving research and make comparisons of carer / caregiver prevalence figures difficult across studies [18]. Although the data were age/sex standardized no adjustment for Consumer Price Index (CPI) was undertaken, so those household income results should be assessed with caution. There was a potential bias from survey non-response in the latter surveys in this study and this should be seen as a weakness of the project. There is a general trend towards lower response rates in all types of population surveys as people protect their privacy, or are overwhelmed by marketing telephone calls or mail outs.

Due to their small numbers, non-English speaking individuals, Indigenous and those from specific cultures are included in the data collection but not in the analysis. Also the small numbers of young carers meant that meaningful analysis could not be included in our study, however this is a limitation that does not detract from their vital roles and urgent need for further research [50–51].

In conclusion, this study demonstrates there has been an overall increase in informal caregiving in SA, a state which over the past two decades has shown higher proportions of people aged 65 years and over, than those in other mainland Eastern Australian states [29]. Our research has highlighted major demographic shifts between 1994 and 2014 and it is important that policy and planning keep pace with these changes and projections. As future prevalence rates are watched with interest in SA, these findings may also be relevant in other specific populations with similar demographic profiles. If we are to sustain the current model of care in the community and the informal carers in their caring role it is important to continue monitoring the prevalence, demographic and health profiles as emerging generations of informal carers with more diverse characteristics take on the caring roles. To achieve this there is an urgent need to include informal carers within ongoing mainstream population surveys, wherever appropriate, so as to provide statistics to plan for their future health care and home support. As the literature suggests, many carers themselves have health problems or suffer diminished quality of life [52–53]. Therefore carer well-being and particularly their health status needs to be considered in parallel with those persons they are caring for. Important as it is to track the prevalence of the wide range of conditions which influence the burden of disease, it is also important to track the prevalence, demographic profiles and health status of those who provide the informal care, as many are carrying a double burden of disease. That of the care recipient, and their own.

## Supporting Information

S1 File Carer Questions (Health Omnibus Surveys 1994–2014). (PDF)

S2 File Socio-demographic Questions (Health Omnibus Surveys 1994–2014). (PDF)
