# Supplementary material for: Unpaid Informal Caregivers in South Australia: Population Characteristics, Prevalence and Age-Period-Cohort Effects 1994–2014
Source: PLoS One. 2016 Sep 20;11(9):e0161994. doi: 10.1371/journal.pone.0161994 (PMC5029908; doi:10.1371/journal.pone.0161994)
Supplement: S1 File — (PDF) [file pone.0161994.s001.pdf]

S1. Carer Questions (Health Omnibus Surveys 1994-2014)

1994: Are you a carer of a dependent person? Yes / No ?
[A dependent person is someone who has a chronic condition that is unlikely to improve.
A carer is responsible for the permanent care of that person on a long term basis.]

1998a: Are you a carer of a dependent person? Yes / No ?
[A dependent person is someone who has a chronic condition that is unlikely to improve.
A carer is responsible for the permanent care of that person on a long term basis.]

2000 and
2001: Do you care at home for a parent, partner, child, other relative or friend who has a disability, is frail aged or who has a chronic mental or physical illness? Yes / No ?

2002: Are you a carer of a dependent person? Yes / No ?
[A dependent person is someone who has a chronic condition that is unlikely to improve.
A carer is responsible for the permanent care of that person on a long term basis.]

2004a: Do you provide long term care at home for a parent, partner, child, other relative or friend who has a disability, is frail aged or who has a chronic mental or physical illness?
Yes / No ?
[Long term care is a minimum of 6 months and may extend into years]

2008a: Do you provide long term care at home for a parent, partner, child, other relative or friend who has a disability, is frail aged or who has a chronic mental or physical illness?
Yes / No ?
[Long term care is a minimum of 6 months and may extend into years]

2009 and
2010: Do you provide any ongoing care or assistance care at home to a relative, friend or neighbour who has a disability, is frail aged or who has a chronic illness including mental illness? Yes / No ?

2013 and
2014: Do you provide long term care at home for a parent, partner, child, other relative or friend who has a disability, is frail aged or who has a chronic mental or physical illness?
Yes / No ?
[Long term care is a minimum of 6 months and may extend into years]
