# Supplementary material for: Unpaid Informal Caregivers in South Australia: Population Characteristics, Prevalence and Age-Period-Cohort Effects 1994–2014
Source: PLoS One. 2016 Sep 20;11(9):e0161994. doi: 10.1371/journal.pone.0161994 (PMC5029908; doi:10.1371/journal.pone.0161994)
Supplement: S2 File — (PDF) [file pone.0161994.s002.pdf]

S2 Socio-demographic Questions (Health Omnibus Surveys 1994-2014)

1. **Sex** : Interviewer recorded gender of the participant
    - Male
    - Female
2. **Age** : As health is often age related may I commence by asking your age?
    All ages were then recoded into the following categories
    - 18 to 49
    - 50 to 69
    - 70 years and over

3. **Area** : Interviewer recorded area of residence – recoded into:
    - Metropolitan
    - Country

4. **Country of Birth:** In which country were you born?
    - Australia
    - UK and Ireland
    - Italy
    - Greece
    - Holland
    - Germany
    - Other European
    - New Zealand
    - African Country
    - Asian Country
    - South America
    - North America
    - Oceania
    - Other (specify)

    The countries were then recoded into the following groups
    - Australia
    - UK/Ireland
    - Other

5. **Marital Status :** What is your marital status?
    - Married
    - De Facto
    - Separated/Divorced
    - Widowed
    - Never Married

    The above marital status categories were recoded into the following groups;
    - Married / defacto
    - Separated/ Divorced
    - Never Married

6. **Educational Attainment:** Which of these groups best describes the highest qualification you have obtained?
    - Still at school
    - Left school at 15 years or less

- Left school after age 15
- Left school after age 15 but still studying
- Trade qualification/ apprenticeship
- Certificate/Diploma – one year full time or less
- Certificate/Diploma – more than one year full time
- Bachelor degree or higher

The above marital status categories were recoded into the following groups;

- Secondary Schooling
- Trade qualifications, Certificate, Diploma
- Bachelor Degree or higher

7. **Employment Status:** Which of these best describes your work status?

- Work full time
- Work part time
- Home Duties
- Unemployed
- Retired
- Student
- Other

The above employment status categories were recoded into the following groups;

- Employed full or part time
- Home Duties
- Retired

8. **Household Annual Income :** I would now like to ask you about your household's income. We are interested in how income relates to lifestyle and access to health services.

Before tax is taken out, which of the following ranges best describes your household's income, from all sources, over the last 12 months?

- Up to $12,000
- $12,001 - $20,000
- $20,001 - $30,000
- $30,001 - $40,000
- $40,001 - $50,000
- $50,001 - $60,000
- $60,001 - $80,000
- $80,001 - $100,000
- $100,001 - $120,000
- $120,001 - $140,000
- $140,001 - $160,000
- $160,001 - $180,000
- $180,001 or more
- Not stated

The above income groups were recoded into the following categories ;

- $40,000 or more
- $20,000 - $40,000
- Less than $20,000
